# Analgesic Effect of Low-Level Laser Therapy before Heel Lance for Pain Management in Healthy Term Neonates: A Randomized Controlled Trial

**DOI:** 10.3390/children10121901

**Published:** 2023-12-08

**Authors:** Bei-Yu Wu, Mei-Chen Ou-Yang, Chun-Ting Liu, Hsin-Chun Huang, Wen-Long Hu, I-Lun Chen, Hsin-Yu Chang, Mei-Yung Chung, Feng-Shun Chen, Yung-Hsiang Chen, Chih-Cheng Chen

**Affiliations:** 1 Department of Chinese Medicine, Kaohsiung Chang Gung Memorial Hospital, Chang Gung University College of Medicine, Kaohsiung 83342, Taiwan; y7802@cgmh.org.tw (B.-Y.W.); juntin0214@cgmh.org.tw (C.-T.L.); oolonghu@cgmh.org.tw (W.-L.H.); 2 Graduate Institute of Integrated Medicine, College of Chinese Medicine, Research Center for Chinese Medicine & Acupuncture, China Medical University, Taichung 404333, Taiwan; 3 College of Nursing, Fooyin University, Kaohsiung 83102, Taiwan; 4 Division of Neonatology, Department of Pediatrics, Kaohsiung Chang Gung Memorial Hospital, Chang Gung University College of Medicine, Kaohsiung 83342, Taiwan; oymj@cgmh.org.tw (M.-C.O.-Y.); hhuang@cgmh.org.tw (H.-C.H.); memeo@cgmh.org.tw (I.-L.C.); b9402050@cgmh.org.tw (H.-Y.C.); chungmy@cgmh.org.tw (M.-Y.C.); fschen@cgmh.org.tw (F.-S.C.); 5 College of Medicine, Kaohsiung Medical University, Kaohsiung 80708, Taiwan; 6 Department of Psychology, College of Medical and Health Science, Asia University, Taichung 413305, Taiwan

**Keywords:** neonate, pain, low-level laser therapy, laser acupuncture

## Abstract

Currently, the prevention, assessment, and management of procedural pain in neonates continues to challenge clinicians and researchers. Objective. To investigate the analgesic effect of low-level laser therapy (LLLT) during heel lance compared to breast milk (BM) feeding in healthy term neonates. In this randomized controlled trial, healthy term neonates who underwent heel lance were randomly assigned to an LLLT or a BM group. The LLLT group received laser therapy to the heel lance site for 20 s before heel lance. The BM group received 5 mL expressed BM via a syringe before heel lance. The primary outcomes were behavioral responses. The secondary outcomes were physiological responses and levels of salivary cortisol and α-amylase. A total of 125 neonates were included, of whom 55 in the LLLT group and 59 in the BM group completed the study. There were no significant differences in latency to first cry and cry duration between the two groups. The squeeze time was significantly shorter in the LLLT group than in the BM group (*p* = 0.047). There were no significant differences in pain scores, heart rate, respiratory rate, oxygen saturation, and blood pressure before and after heel lance between the two groups. There were no significant differences in salivary cortisol and α-amylase levels in the LLLT group before and after heel lance; however, the differences were significant in the BM group. These findings suggest that the analgesic effect of LLLT is similar to that of BM during heel lance in healthy term neonates. LLLT has potential as an analgesic treatment.

## 1. Introduction

Routine newborn screening and intensive care frequently cause anticipated procedural pain. The American Academy of Pediatrics (AAP) advocates preventing and minimizing pain in neonates, and that unavoidable pain should be treated promptly and adequately [1]. Nevertheless, neonatal pain is often ignored and inadequately treated. A large prospective study conducted in France reported that a total of 58.2% of heel lances were performed with specific preprocedural analgesia [2]. Healthy neonates routinely undergo heel lance procedures. Heel lance, also called heel stick, is an invasive procedure during which a neonate’s heel is pricked to obtain blood shortly after birth for screening tests or measurements of serum bilirubin or glucose. The pricked site is on the inner or outer sole surface of the heel. The heel lance procedure is an easily accessible way of obtaining capillary blood, but it is a painful procedure. Heel lance has been demonstrated to be more painful than venipuncture for neonates [3]. The procedural pain is associated with actual heel lancing itself and subsequent heel squeezing. Neonates hospitalized for medical conditions such as jaundice require additional heel lance procedures, and repeated painful stimuli may cause physiological and behavioral responses [4,5]. In addition, early pain experiences may alter pain responses and impact neurodevelopment in later infancy [4,5].

Assessment of pain in neonates is difficult because they are nonverbal. Pain can lead to immediate physiological and behavioral responses. To date, no single physiological or behavioral measure has been identified as a pure pain measure in neonates. It is often necessary to use several assessment tools with multidimensional aspects for an accurate measure of neonatal pain [6]. Physiological pain responses commonly monitored in neonates include heart rate, respiratory rate, oxygen levels, blood pressure, and cortisol levels [6]. Behavioral pain responses commonly monitored include facial expressions, cries, attempts to withdraw the limb, and agitation. The facial activity is starkly visible in infants reacting to noxious events [7]. The Neonatal Facial Coding System (NFCS) is commonly used to examine facial expressions in infants and has been identified as responsive to painful events. The Neonatal Pain, Agitation, and Sedation Scale (NPASS) is recommended by the AAP to measure neonatal pain and sedation [8]. The NPASS consists of both physiological and behavioral pain response measures and has reported to have good reliability and validity for most of the neonatal population [9]. 

In current clinical practice, non-pharmacological interventions are more commonly used for reducing procedural pain than pharmacological treatments due to concerns of adverse events. Previous literature reviews showed that breastfeeding and oral sweet solutions such as sucrose and glucose are effective interventions in pain prevention for neonates during heel lance without serious adverse effects [10,11,12,13,14]. The minimally effective dose of sucrose for procedural pain relief in neonates was reported as 0.1 mL [15]. Adverse effects of breastfeeding and oral sweet solutions are rare and may include choking, transient bradycardia, and oxygen desaturation in sick or extremely preterm infants [16]. Benoit and colleagues reported that breastfeeding was as or more effective than sweet solutions in full-term neonates [13]. Nevertheless, clinicians may encounter challenges in selecting appropriate analgesic interventions for neonates because of mothers unable to breastfeed in specific conditions such as prenatal and labor-related complications. In addition, the impacts of long-term or repeated sucrose administration on the neurodevelopment of infants particularly in preterm infants are still not fully understood [1,13]. Therefore, a more feasible and effective intervention to reduce procedural pain during heel lance is needed. 

Low-level laser therapy (LLLT), also known as photobiomodulation, is the application of light with a wavelength in the red to near infrared region. LLLT has been shown to reduce pain, inflammation, and edema, and to prevent tissue damage [17,18]. The use of LLLT for pain management is increasing in adults and children because it is a safe, non-invasive, and painless nonpharmacologic technique [19]. LLLT induces analgesia by blocking the conduction of central and peripheral nerve fibers and the release of endorphins [20,21]. LLLT could suppress afferent fiber signaling and modulate synaptic transmission to dorsal horn neurons, including inhibition of substance P, which may result in long-term pain suppression [21]. Before pain or tissue damage occurs, LLLT pretreatment to normal cells or tissue can also elicit a protective response [22]. LLLT pretreatment has been shown to improve muscle fatigue and exhaustion via decreasing inflammatory biomarkers and lactate levels after exercise [22]. Additionally, LLLT pretreatment can exhibit anti-nociceptive and anti-inflammatory effects in animal models via enhancing peripheral endogenous opioid production and reducing inflammatory cells, respectively [22,23]. However, the mechanism of LLLT pretreatment is still not fully understood. Few studies have investigated the application of LLLT for pain management in neonates during heel lance. One study, which used laser acupuncture for pain management in healthy term newborns before heel lance, reported negative results compared with oral sucrose [24]. In that study, LLLT involved 0.3 J of energy to the Yintang (EX-HN3) acupoint located on the face at the midpoint between the medial ends of the eyebrows at a wavelength of 905 nm and a frequency of 73 Hz [24]. For LLLT to be effective, these parameters are important; however, the optimal values including power output, wavelength, frequency, and duration of stimulation remain controversial. 

The knowledge of pain management in neonates has increased dramatically in the past three decades; however, gaps exist in knowledge, evidence, and practice in neonatal pain assessment and management [25]. In order to find a safe, effective, and more feasible method for the management of procedural pain in neonates, we aimed to investigate the analgesic effect of LLLT pretreatment to the heel lance site with different parameters. We hypothesized that LLLT could reduce nociceptive pain during heel lance in neonates as well as BM feeding can, which, in turn, could reduce physiological and behavioral responses to procedural pain. This study can verify whether LLLT has potential for pain management in neonates, in the hope that this can improve the knowledge-to-practice gap in neonatal pain care.

## 2. Materials and Methods

### 2.1. Study Design, Setting, Sample Size, Blinding, and Randomization

**Figure 1:**
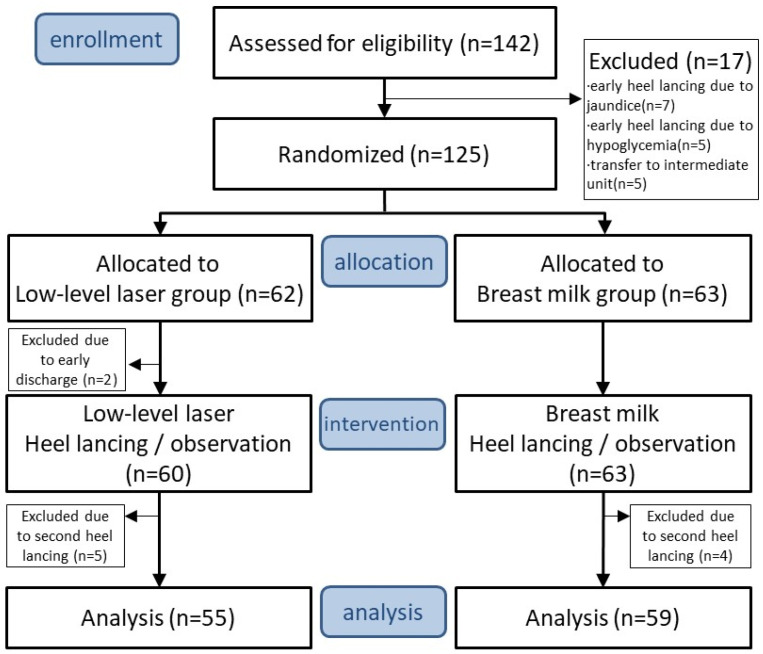
A flowchart of the trial.

This prospective, single-center, open-label, randomized controlled trial was performed at the newborn nursery of Kaohsiung Chang Gung Memorial Hospital (KCGMH), a tertiary medical center in Taiwan, from August 2017 to October 2018. Healthy term newborns hospitalized in the newborn nursery were recruited. The eligibility of participants’ parents who expressed interest in the study was assessed through a review of their medical records and an interview conducted by a neonatologist at KCGMH. The participants who satisfied the inclusion criteria were allocated at random to either an LLLT group or a BM group. The study design according to the CONSORT 2010 is depicted in Figure 1. Written informed consent was obtained from the participants’ parents before randomization.

#### 2.1.1. Sample Size Calculation

Determination of the necessary sample size was based on a previous study [26] on the analgesic effects of glucose water for procedural pain in preterm neonates. One-min pain scores were 5.83 ± 2.77 in the glucose group and 7.66 ± 3.24 in the control group. Anticipating a power of 90% (1 − β = 0.9), statistical significance (α = 0.05) of 95%, and a dropout rate of 10% using G Power software (G*Power 3.1.9.2, Heinrich-Heine-Universität Düsseldorf, Düsseldorf, Germany), a total of 130 participants were required in this study.

#### 2.1.2. Randomization

The participants were randomly assigned to the LLLT group or the BM group at a ratio of 1:1. For allocation of the participants, we used computer-generated random numbers from Excel^®^ (version 2010, Microsoft Office, Redmond, WA, USA). The randomization process utilized block randomization with two treatment groups and a block size of 2. An independent researcher, Feng-Shun Chen, who was not involved in the inclusion or exclusion process, treatment, or assessment procedures, conducted the randomization procedure. The randomization sequences were contained in a set of sealed envelopes before allocation. Before the heel lance procedure, the physician and the research nurse did not know which group the participants were assigned to until the research nurse opened the envelope and informed the physician.

#### 2.1.3. Blinding

This study was designed as an open-label study since it involved healthy neonates, as they had a similar physiological state and were unaware of the study process before heel lance. The physician who performed the LLLT and the research nurse who administered the BM and performed the heel lance procedures were not involved in the clinical care in the newborn nursery. The research nurse and the physician knew the treatment allocation but were not involved in the outcome assessment. The outcomes were assessed by another three investigators (I-Lun Chen, Hsin-Yu Chang, and Mei-Yung Chung) who were blinded to treatment allocation. 

### 2.2. Participants

The eligibility of participants to take part in the study was evaluated by neonatologists. All the participants received standard care by the neonatology team from the time of admission. The criteria for inclusion in the study were as follows: (1) term birth between 37 and 42 weeks of gestation; (2) an Apgar score of 7 or more at 5 min after birth; (3) No signs of illness, or abnormalities in a physical examination. The participants with the following specified conditions were excluded: (1) an Apgar score of less than 7 at 5 min after birth; (2) birth injuries; (3) major malformations; (4) severe congenital anomalies; (5) cyanotic heart disease; (6) severe infectious diseases; (7) drug withdrawal; (8) previous treatment with analgesics or sedatives; (9) any other diseases/conditions requiring neonatal intensive care. Furthermore, participants would be dropped from the study based on the following conditions: (1) crying for more than 5 min before the heel lance procedure; (2) need for a second heel lance during the study intervention; (3) voluntary decision by the participants’ parents to withdraw from the trial at any time.

It must be added that the heel lance in this study needed to be the first heel lance in the included cases. The participants were excluded if they needed a second heel lance due to insufficient blood collection. The participants were also excluded due to early heel lance before the routine time due to jaundice or hypoglycemia.

### 2.3. Intervention

**Figure 2:**
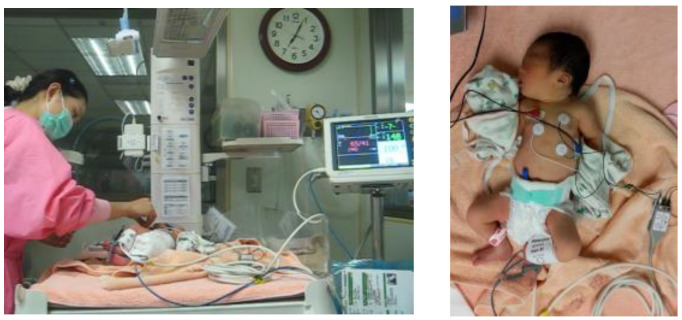
Illustration of how research participants were photographed. (**left**) One camera recorded the EKG monitor and the procedural time. (**right**) The other camera recorded the participants’ behavior for crying time, NPASS, and NFCS.

All participants in both the LLLT and BM groups were studied in an isolated quiet room. They were required to have been fed within 3 h but not within 30 min before the study. The participants were placed on an infant warmer (IW934 Baby Control CosyCot™ Infant Warmer, Auckland, New Zealand) which was set in prewarm mode. A patient monitor (SureSigns VM4, Koninklijke Philips N.V.) was used to assess heart rate, respiratory rate, blood pressure, and oxygen saturation throughout the study process. During filming, two digital cameras were used in this study. One (the left Figure 2) continuously recorded the monitor, and the other (the right Figure 2) continuously recorded the participants’ behavior. We started the recording before baseline, then continued it throughout the study process and usually finished it when the baby stopped to cry after heel lance. The length of the video was around 8–10 min.

**Figure 3:**
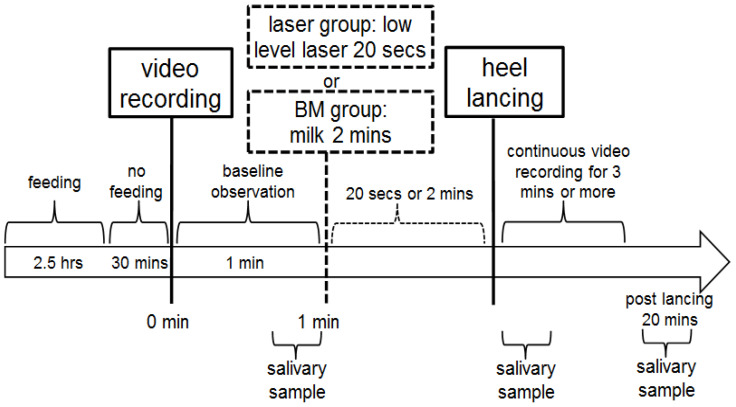
The intervention protocol in this study.

Salivary samples were obtained at baseline, and immediately and 20 min after heel lance. Digital cameras continuously recorded the monitor and the participants’ behavior throughout the study process. Parents were not allowed into the room where the heel lance took place. Two observers (I-Lun Chen, Hsin-Yu Chang), who were blinded to the allocation and intervention, reviewed the videos and assessed the data on patient monitors, crying time, and pain scores. Another investigator (Mei-Yung Chung) collected and assessed the data, and then confirmed the final value of each variable. The intervention protocol did not involve the parents. The intervention protocol is shown in Figure 3.

#### 2.3.1. Low-Level Laser Therapy (LLLT) Group

After collecting baseline saliva samples, the participants in the LLLT group received LLLT to the heel lance site using a LaserPen (power 150 mW, wavelength 810 nm, and Nogier frequency E 4672 Hz; RJ-laser, Waldkirch, Germany) for 20 s (equal to 1.5 J of energy) by the same physician. After LLLT, heel lance was performed by the same research nurse. 

#### 2.3.2. Breast Milk (BM) Group

After collecting baseline saliva samples, the participants in the BM group were given 5 mL expressed BM via a syringe over a 2 min period. After milk feeding, heel lance was performed by the same research nurse. 

### 2.4. Outcome Measurements

There is no gold standard to assess pain in neonates as their responses to pain are nonverbal. In the present study, both behavioral and physiological responses were measured to ensure an accurate measure of neonatal pain. The primary outcomes were behavioral responses during the heel lance procedure including the latency to first cry, duration of crying, squeeze time, and Neonatal Pain Agitation and Sedation Scale (NPASS) and Neonatal Facial Coding System (NFCS) scores. The secondary outcomes were physiological responses during the heel lance procedure including changes in heart rate, respiratory rate, oxygen saturation, and blood pressure (systolic and diastolic). In addition, the levels of salivary cortisol and α-amylase were measured as pain-related stress biomarkers. Behavioral and physiological responses were recorded throughout the study process by separate digital cameras that were focused on the neonate’s face and body and the monitor.

#### 2.4.1. Assessment of Behavioral Outcomes

The latency to first cry was defined as the time interval between the heel lance and when the neonate started to cry. The duration of crying was defined as the time interval between the start of crying and the end of crying (silence for >5 s). Squeeze time was defined as the total collection time counted from the moment the lancet punctured the neonate’s foot to the end of the collection of five capillaries of blood. Pain was assessed before and at 1, 2, and 3 min after heel lance using the NPASS and the NFCS. The NPASS is based on five criteria to assess pain in neonates: crying/irritability, behavior, facial expression, extremity tone, and vital signs. Each criterion is graded on a 0–2 scale. Scores for each criterion are summed for a total pain score of 0–10, with a higher score indicating greater pain [27]. The NFCS is used to examine facial activity in neonates to assess pain. It contains eight facial actions: wrinkled forehead, compressed palpebral fissures, deep nasolabial groove, half open lips, mouth vertically or horizontally outstretched, tense tongue, tongue protrusion, and quivering of the chin. Each facial action is graded as 0 if absent or 1 if observed. Scores on each facial action are summed for a total pain score of 0–8, with a higher score indicating greater pain [28].

#### 2.4.2. Assessment of Physiological Outcomes

Heart rate, respiratory rate, and oxygen saturation were recorded every 15 s, and the data collected over 1 min before treatment were averaged to determine the baseline values. In addition, the data collected over 3 min after heel lance were averaged to determine the values after the procedure. Blood pressure was assessed before and 3 min after heel lance.

#### 2.4.3. Analysis of Salivary Stress Biomarkers

Salivary samples were collected using SalivaBio Infant’s Swab (SIS) (Salimetrics^®^, State College, PA, USA) according to the manufacturer’s instructions. The samples were obtained from the participants before and immediately and 20 min after heel lance. After collection, the specimens were promptly refrigerated at 4 °C to prevent bacterial proliferation. Within 4 h of collection, the samples were further preserved at −20 °C until analysis. Prior to analysis, the samples underwent complete thawing, vortexing, and centrifugation at 1500× *g* for 15 min to eliminate mucins and other particulate matter that could potentially interfere with the assay and impact the results.

Salivary cortisol levels were assessed using a Salimetrics^®^ Cortisol Enzyme Immunoassay Kit (Item No. 1-3002, Salimetrics LLC, State College, PA, USA), a competitive immunoassay designed for the quantitative measurement of salivary cortisol. The assay involves the competition of cortisol in standards and samples with cortisol conjugated to horseradish peroxidase for the antibody binding sites on a microtiter plate. Following the manufacturer’s protocol, unbound components were removed after incubation, and the bound cortisol enzyme conjugate was quantified through the reaction of horseradish peroxidase enzyme with the substrate tetramethylbenzidine. This reaction generates a blue color, which turns yellow upon the addition of an acidic solution to stop the reaction. The optical density was then measured at 450 nm using a standard plate reader. 

Salivary α-amylase activity was assessed utilizing a Salimetrics^®^α-Amylase Kinetic Enzyme Assay Kit (Item No. 1-1902, Salimetrics LLC, State College, PA, USA). Amylase activity was assessed in accordance with the manufacturer’s protocol. Salivary samples were diluted 50~200 fold with α-Amylase Diluent, heated substrate was added to start the reaction, and the optical density was read at exactly 1 and 3 min. The α-amylase activity was calculated at 1 min and 3 min and multiplied by the conversion factor. All samples were analyzed in duplicate for cortisol and α-amylase.

### 2.5. Ethics Approval

The protocol was registered with ClinicalTrials.gov (Identifier: NCT03268148). The study was approved by the Human Ethics Committee of Chang Gung Medical Foundation Institutional Review Board, IRB No. 201700474A3C501 (the approval date was 19 July 2017). This study was carried out in compliance with the principles of the Declaration of Helsinki. Both verbal and written forms of detailed information about the trial were provided by neonatologists at KCGMH before participation. All parents of the participants voluntarily signed and provided informed consent, which had been approved by the ethics committee, prior to their enrollment. The study participants did not receive any monetary compensation and were informed of their right to withdraw from the study at any point. Information regarding potential and enrolled participants’ personal details was gathered, exchanged, and stored in a separate and secure storage space to safeguard the confidentiality of the participants throughout the trial process.

### 2.6. Statistical Analyses

All analyses were performed using SPSS 22.0 for Windows (Statistics 22.0, SPSS, IBM, New York, NY, USA). A chi-square test was used to compare the demographic data including gender and delivery type. An independent t test was used to compare the demographic data including gestation, birth weight, and Apgar score, and primary/secondary outcome variables between the LLLT and BM groups. In addition, a paired t test was used to compare salivary cortisol and salivary amylase levels before and after the procedure in each group. Differences with a *p* value of less than 0.05 were considered to be statistically significant in all analyses.

### 2.7. Data Monitoring

Data Monitoring Committee (DMC) approval was not required because laser acupuncture and BM feeding are general practice and noninvasive interventions. However, all participants were recorded to monitor any adverse events which may have occurred after the intervention, such as vomiting, local redness, and swelling of the lance site. 

## 3. Results

During the study period, 142 neonates were assessed for eligibility and a total of 125 neonates were recruited and randomly assigned to the LLLT group (n = 62) and the BM group (n = 63) from August 2017 to October 2018. All participants’ parents agreed to participate in the study and signed the informed consent. Two neonates in the LLLT group withdrew from the study because of early discharge before the intervention. Five neonates in the LLLT group and four neonates in the BM group were excluded due to insufficient blood collection and because they needed a second heel lance during the study intervention. A total of 55 neonates in the LLLT group and 59 neonates in the BM group were included for analysis. The study flowchart is shown in Figure 1. The demographic characteristics were similar between the two groups (Table 1). There were no significant differences between the two groups in gestational age (39.31 ± 1.02 vs. 39.10 ± 1.18, *p* = 0.299), birth weight (3130.91 ± 368.15 vs. 3122.63 ± 370.61, *p* = 0.905), gender (male, 40% vs. 49.2%), delivery type (Cesarian, 23.6% vs. 25.4%), and Apgar score (1 min, 9 ± 0 vs. 9 ± 0, *p* = 1; 5 min, 10 ± 0 vs. 10 ± 0, *p* = 1).

### 3.1. Primary Outcomes

**Figure 4:**
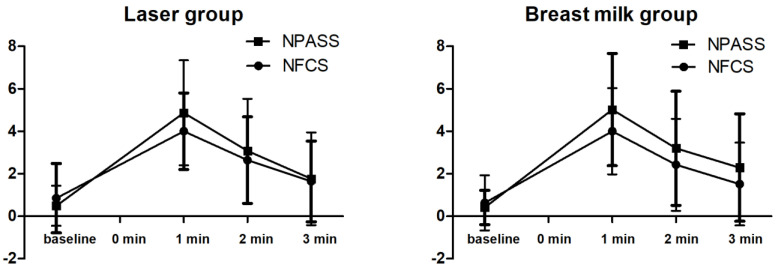
Changes in pain scores (NPASS and NFCS) at baseline and 1, 2, and 3 min after heel lance. Data are presented as mean ± SEM.

The behavioral responses are shown in Table 2. There was no significant difference in the latency to first cry between the two groups (mean ± SD; 5.61 ± 12.09 and 5.10 ± 7.27, *p* = 0.795). The duration of crying was shorter in the LLLT group than in the BM group, but the difference was not statistically significant (mean ± SD; 97.07 ± 62.16 and 128.08 ± 108.16, respectively, *p* = 0.062). The squeeze time was significantly shorter in the LLLT group than in the BM group (mean ± SD; 95.07 ± 40.16 and 122.51 ± 95.98, respectively, *p* = 0.047). There were no significant differences in the NPASS and the NFCS pain scores between the two groups at baseline and at 1, 2, and 3 min after heel lance (Figure 4).

### 3.2. Secondary Outcomes

**Figure 5:**
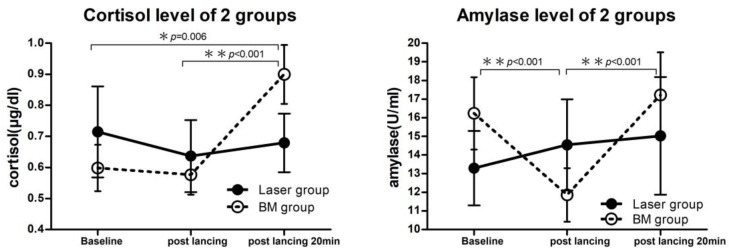
Changes in salivary cortisol and amylase levels before and after heel lance. Data are presented as mean ± SEM.

Physiological parameters including heart rate, respiratory rate, oxygen saturation, and blood pressure were monitored before and after heel lance. There were no significant differences in these parameters between the two groups (Table 3). Regarding the measurements of salivary cortisol and α-amylase, the volume of one specimen collected in the BM group was insufficient for analysis, and the volumes of four specimens collected in the LLLT group were only sufficient to analyze cortisol levels. The results of salivary cortisol and α-amylase levels are shown in Table 4 and Table 5. There were no significant differences in salivary cortisol and α-amylase levels between the two groups before and immediately and 20 min after heel lance. However, Figure 4 and Table 4 show that the salivary cortisol level in the BM group was significantly higher 20 min after heel lance compared to baseline (*p* = 0.006) and immediately after heel lance (*p* < 0.001). In addition, Figure 5 and Table 5 show that the salivary α-amylase level in the BM group was significantly lower immediately after heel lance compared to baseline (*p* < 0.001), and the level was significantly higher 20 min after heel lance compared to immediately after heel lance (*p* < 0.001). On the contrary, there were no significant differences in salivary cortisol and α-amylase levels in the LLLT group before and immediately and 20 min after heel lance compared to baseline.

### 3.3. Adverse Events

Participants were not visibly uncomfortable while receiving LLLT. No adverse events after the intervention such as vomiting, local redness, and swelling at the lancing site were reported throughout the trial.

## 4. Discussion

The main findings of the present study are that there was a shorter procedure time in the LLLT group compared to the BM group, and that there were no significant differences between the two groups in latency to first cry, crying duration, and NAPSS/NFCS scores. In addition, there were no significant differences in the physiologic stress biomarkers salivary cortisol and α-amylase between the LLLT and BM groups. 

According to the 2016 update on the management and prevention of procedural pain in neonates from the AAP, breastfeeding during heel lance substantially reduces the pain responses in term neonates compared to positioning, rocking, and maternal holding [1]. This may be related to the sweetness of BM, the high concentration of beta-endorphins, and/or skin-to-skin contact between mother and baby while breastfeeding [29]. The AAP guideline, and later Cochrane systemic reviews, suggested that breastfeeding and sweet solutions are effective to reduce procedural pain in neonates [1,11,12,13,14]. The AAP guideline [1] and other studies also recommended other nonpharmacologic strategies such as non-nutritive sucking, provision of expressed BM, or skin-to-skin contact, which have been shown to be useful in reducing procedural pain and should be consistently used [25]. Nevertheless, in clinical practice, there may be some barriers to breastfeeding before or during a painful procedure. For example, the mother is unwilling to breastfeed. Breastfeeding might be unsuitable or inadequate due to lactation problems or other medical conditions. In theory, parents are welcome to be a part of this process by holding their baby while the heel lance is performed; however, in practice, the heel lance is performed in the early morning approximately between 5 am and 6 am in our hospital, because the specimens need to dry completely, which may need 3 to 4 h before we sent the specimens to the laboratory. The specimens transport time may need another 3 to 4 h. In order to allow mothers to have enough sleep and complete the examination in time, parents are usually not involved to the heel lance procedure. In addition, exclusive breastfeeding is usually recommended as the ideal food for neonates in Taiwan [30]. Exclusive breastfeeding means that the neonates receive only breast milk without any other liquids or solids. According to a 2016 national survey in Taiwan, the exclusive breastfeeding rate during the first month after delivery reached 66% [30]. In recent years, the Taiwanese government has continued to encourage exclusive breastfeeding. Most people in modern Taiwan believe that BM is the best food for neonates, and they are relatively opposed to feeding neonates with other sweet solutions. Due to the above factors and based on our prior study, feeding with expressed BM via a syringe is the most common strategy used to reduce pain before heel lance in our hospital. Our prior study showed expressed BM is similarly effective as 25% glucose in reducing pain scores during heel lance in preterm neonates [26]. Hsieh and colleagues also reported that BM or 10% dextrose water is safe and effective in reducing the procedural pain, and that BM is the priority [31]. 

Although LLLT is generally used for pain management, the application of LLLT to prevent pain during heel lance in neonates is still unproven. One previous study used a thin (0.22 × 1.5 mm) sterile disposable needle at the Yintang acupoint for 30 min due to its reported effects of sedation and analgesia according to the theory of acupuncture [32]. The results showed that crying time and pain scores during heel lance were lower in the preterm neonates who received BM combined with acupuncture compared to BM alone [32]. However, to the best of our knowledge, only one previous study has investigated the use of LLLT [24]. In that study, 0.3 J of laser energy applied to the Yintang acupoint before heel lance was less effective than oral sucrose in reducing discomfort during heel lance [24]. The main findings of the study were a shorter procedure time but longer crying duration and higher pain score in the LLLT group compared to the oral sucrose group [24]. The effects of acupuncture and LLLT to the Yintang acupoint to reduce pain in previous studies are therefore inconsistent. Possible reasons include diffuse nonspecific effects caused by acupuncture and ineffective LLLT parameters. In addition, the choice of treatment location is also important. The location of the Yintang acupoint is far away from the site of the heel lance. Another study reported that acupressure at the Kunlun (BL60) and Taixi (KI3) acupoints before heel lance was associated with a shorter procedural time and shorter crying duration in preterm neonates [33]. The Kunlun and Taixi acupoints are located posterior to the lateral and medical malleolus and are very close to the site of the heel lance. We think that the analgesic effect of applying acupressure on the Kunlun and Taixi acupoints may be associated with a conduction block of lateral calcaneal branches of sural nerves and medical calcaneal branches of tibial nerves. In addition, we believe that the analgesic effect of LLLT to the heel lance site can be produced sooner and more effectively via conduction block of surrounding nerves compared to distant locations. Therefore, in the present study, we applied LLLT to the heel lance site. We also used a different laser frequency and increased the energy to 1.5 J compared to the previous study. Our findings showed that LLLT had similar behavioral and physiological responses and a shorter procedure time compared to BM.

In the present study, we evaluated the physiological responses to pain from heel lance by measuring heart rate, respiratory rate, and oxygen saturation as well as levels of salivary cortisol and α-amylase. The salivary cortisol level has been reported to be a reliable tool to evaluate pain in neonates, and its concentration measured 25 min after a painful procedure is typically significantly higher compared to baseline [34]. Salivary α-amylase is also a valid and reliable biomarker to evaluate stress and pain in adults; however, its applicability is limited in neonates because maturation of the salivary glands is not achieved until later in infancy [35]. In the present study, the salivary cortisol and α-amylase levels in the LLLT group were not significantly different before and after heel lance. In contrast, the salivary cortisol level in the BM group showed a significant increase 20 min after heel lance compared to baseline. This finding suggests that the neonates in the BM group had a higher stress response to pain. A previous study reported that salivary α-amylase was not suitable to assess pain in newborns during heel lance due to no differences in response to stress [36]. However, we found that the α-amylase level measured immediately after heel lance was significantly decreased in the BM group compared to baseline. The reason for this finding is not clear, but it may be due to high variability in both basal α-amylase activity and α-amylase responses to stress among different ages, salivary gland development, stress levels, and even eating habits [37,38].

There are several limitations to our study. First, the study was conducted at a single medical center. In addition, an analgesic effect may have occurred due to pressure on the skin by the laser probe. However, in order to avoid this situation, the physician was asked to apply a light touch on the skin during LLLT. Therefore, we believe that an analgesic effect caused by pressure from the laser probe can be neglected. Furthermore, researchers may have ethical concerns about the choice of expressed BM as an active comparator instead of breastfeeding or sweet solutions as a standard of care. Currently, randomized controlled trials are regarded as the gold standard trial for evaluating the effectiveness of interventions and must comply with ethical requirements. The Declaration of Helsinki is the most widely accepted set of ethical principles in medical research. According to the paragraph on placebo use in the Declaration of Helsinki, the use of any intervention less effective than the best proven one, the use of placebo, and no intervention are accepted and permitted when there are compelling and scientifically methodological reasons, the research is intended to determine the efficacy or safety of an intervention, and the patients who receive any intervention less effective than the best proven one, placebo, or no intervention are not at risks of serious or irreversible harm as a result of not receiving the best proven intervention [39]. In the present study, we aimed to determine the analgesic effect of LLLT during a single heel lance procedure. The participants’ parents were adequately informed about the potential risks during enrollment and have therefore knowingly consented. The participants may experience more pain if LLLT is not effective, but we think that there are no additional risks of serious or irreversible harm for neonates during a single procedure. Therefore, it is our position that the research design is justified.

Although breastfeeding or sweet solutions are currently considered effective methods for procedural pain in neonates, it is difficult to translate the knowledge and implement best evidence to clinical practice. The possible reasons include a lack of time, ineffective continuing education, increasing complexity of medical procedures and treatments, inadequate application of evidence to case management, and lack of adequate communication between researchers and policy makers [40]. In addition, the best available evidence and recommendations are not suitable for all health-care settings, particularly in low-income and middle-income countries [41,42]. Continuing education, globally accepted pain management guidelines, and readily applied pain management tools would further bridge the knowledge–practice gap related to neonatal pain management [43].

## 5. Conclusions

The present study showed that healthy term neonates receiving LLLT before heel lance had similar physiological and behavioral responses compared to feeding with BM as well as a shorter procedural time and less variability in salivary cortisol and α-amylase. These findings suggest that the analgesic effect of LLLT is at least as effective as BM for procedural pain during heel lance in healthy term neonates. In a clinical setting, the administration of LLLT to full-term neonates should be considered to reduce procedural pain associated with heel lance. LLLT has potential for future development as an analgesic treatment.

## Figures and Tables

**Table 1:** Demographics and clinical characteristics of the neonates by treatment group ^1^.

	LLLT ^2^ Group (n = 55)	BM ^2^ Group (n = 59)	*p* Value
Gestation (weeks)	39.31 ± 1.02	39.10 ± 1.18	0.299
Birth weight (g)	3130.91 ± 368.15	3122.63 ± 370.61	0.905
Gender			
Male	22 (40.0)	29 (49.2)	0.326
Female	33 (60.0)	30 (50.8)	
Delivery type			
Cesarian	13 (23.6)	15 (25.4)	0.825
Vaginal	42 (76.4)	44 (74.6)	
Apgar score 1	9 ± 0	9 ± 0	1
Apgar score 5	10 ± 0	10 ± 0	1

^1^ Data are presented as mean ± standard deviation or number (%). ^2^ Abbreviations: LLLT, low-level laser therapy; BM, breast milk.

**Table 2:** Behavioral responses after heel lance of neonates by treatment group ^1^.

Time (s)	LLLT ^2^ Group (n = 55)	BM ^2^ Group (n = 59)	*p* Value
Latency to first cry	5.61 ± 12.09	5.10 ± 7.27	0.795
Duration of crying	97.07 ± 62.16	128.08 ± 108.16	0.062
Squeeze time	95.07 ± 40.16	122.51 ± 95.98	0.047 *
NFCS			
Baseline	0.85 ± 1.63	0.63 ± 1.30	0.413
1 min	4.00 ± 1.80	4.00 ± 2.03	1.000
2 min	2.64 ± 2.04	2.42 ± 2.17	0.591
3 min	1.64 ± 1.90	1.51 ± 1.95	0.724
NPASS			
Baseline	0.49 ± 0.94	0.41 ± 0.81	0.609
1 min	4.87 ± 2.47	5.02 ± 2.64	0.764
2 min	3.07 ± 2.46	3.20 ± 2.69	0.787
3 min	1.76 ± 2.19	2.29 ± 2.53	0.240

^1^ Data are presented as mean ± standard deviation. * Indicates *p* < 0.05. ^2^ Abbreviations: LLLT, low-level laser therapy; BM, breast milk; NFCS, Neonatal Facial Coding System; NPASS, Neonatal Pain Agitation and Sedation Scale.

**Table 3:** Physiological parameters of the neonates at baseline and after heel lance by treatment group ^1^.

	LLLT ^2^ Group (n = 55)	BM ^2^ Group (n = 59)	*p* Value
Heart rate (/min)			
Baseline	147.10 ± 21.36	150.61 ± 18.10	0.344
After	163.37 ± 19.09	166.03 ± 18.72	0.640
Respiratory rate (/min)			
Baseline	43.46 ± 11.00	40.18 ± 9.21	0.086
After	45.02 ± 8.49	45.01 ± 9.42	0.999
SaO_2_ (%)			
Baseline	96.77 ± 5.09	95.69 ± 5.47	0.282
After	93.97 ± 5.09	94.89 ± 4.23	0.296
SBP (mmHg)			
Baseline	66.47 ± 9.00	68.12 ± 10.61	0.377
After	68.57 ± 9.23	69.57 ± 9.04	0.566
DBP (mmHg)			
Baseline	41.13 ± 9.31	41.30 ± 9.58	0.924
After	42.89 ± 10.90	40.93 ± 8.50	0.290

^1^ Data are presented as mean ± standard deviation. ^2^ Abbreviations: LLLT, low-level laser therapy; BM, breast milk; SaO_2_, oxygen saturation; SBP, systolic blood pressure; DBP, diastolic blood pressure.

**Table 4:** Salivary cortisol levels of the two study groups ^1^.

Cortisol (μg/dL)	LLLT ^3^ Group (n = 55)	BM ^3^ Group (n = 58)	*p* Value
Baseline	0.71 ± 1.09	0.60 ± 0.57	0.473
Post heel lancing	0.64 ± 0.86	0.58 ± 0.49	0.645
†*p* value ^2^	0.175	0.725	
20 min after heel lancing	0.68 ± 0.70	0.90 ± 0.72	0.103
†*p* value ^2^	0.809	0.006 *	

^1^ Data are presented as mean ± standard deviation. ^2^ †*p* values: compared with the baseline in each group using a paired *t*-test. *p* value: comparing the LLLT group and BM groups using an independent *t*-test. * Indicates *p* < 0.05. ^3^ Abbreviations: LLLT, low-level laser therapy; BM, breast milk.

**Table 5:** Salivary amylase levels of the two study groups ^1^.

Amylase (U/mL)	LLLT ^3^ Group (n = 51)	BM ^3^ Group (n = 58)	*p* Value
Baseline	13.29 ± 14.24	16.24 ± 14.78	0.293
Post heel lancing	14.54 ± 17.49	11.86 ± 10.92	0.333
†*p* value ^2^	0.451	<0.001 *	
20 min after heel lancing	15.03 ± 22.57	17.22 ± 17.47	0.569
†*p* value ^2^	0.374	0.376	

^1^ Data are presented as mean ± standard deviation. ^2^ †*p* values: compared with the baseline in each group using a paired *t*-test. *p* value: comparing the LLLT and BM groups using an independent *t*-test. * Indicates *p* < 0.05. ^3^ Abbreviations: LLLT, low-level laser therapy; BM, breast milk.

## Data Availability

The data presented in this study are available on request from the corresponding author. The data are not publicly available due to their containing information that could compromise the privacy of research participants.
